# Changing patterns of first e-cigarette flavor used and current flavors used by 20,836 adult frequent e-cigarette users in the USA

**DOI:** 10.1186/s12954-018-0238-6

**Published:** 2018-06-28

**Authors:** Christopher Russell, Neil McKeganey, Tiffany Dickson, Mitchell Nides

**Affiliations:** 1 Centre for Substance Use Research, 4.04 West of Scotland Science Park, Kelvin Walkway, Glasgow, G20 0SP UK; 2 Los Angeles Clinical Trials, 4116 W. Magnolia Blvd, Suite 100, Burbank, CA 91505 USA

**Keywords:** E-cigarettes, Flavors, E-liquids, Tobacco, Smoking, Cigarettes, Harm reduction, Vapers, Vaping

## Abstract

**Background:**

Understanding the role that flavors play in the population’s use of e-cigarettes and the impact that flavored e-cigarette products have on the population’s use of more harmful tobacco products, like conventional cigarettes, has been identified by the US Food and Drug Administration (FDA) as a public health research priority. The purpose of the study was to assess the first e-cigarette flavor and current e-cigarette flavors used by a large non-probabilistic sample of adult frequent users of e-cigarettes in the USA and assess how flavor preferences vary by cigarette smoking status and time since first e-cigarette purchase.

**Methods:**

An online survey assessed the first e-cigarette flavor and current e-cigarette flavors used by a non-probabilistic sample of 20,836 adult frequent e-cigarette users in the USA. Differences in e-cigarette flavor preferences between current smokers, former smokers, and never-smokers and trends in the first flavor used across time of e-cigarette use initiation were assessed.

**Results:**

The majority (*n* = 15,807; 76.4%) of sampled frequent e-cigarette users had completely substituted e-cigarettes for conventional cigarettes—“switchers”—and were currently using rechargeable, refillable vaping devices. Among them, the proportion of first e-cigarette purchases that were fruit-flavored increased from 17.8% of first purchases made before 2011 to 33.5% of first purchases made between June 2015 and June 2016. Tobacco-flavored first purchases almost halved during this time (46.0% pre-2011 to 24.0% between 2015 and 2016). Fruit/fruit beverage (73.9 to 82.9% of sampled users), dessert/pastry (63.5 to 68.5% of sampled users), and candy, chocolate, or sweets (48.7 to 53.4% of sampled users) were the most popular currently used e-cigarette flavors. Tobacco and menthol flavors, the two most popular flavors for initiating e-cigarette use prior to 2013, now rank as the 5th and 6th most popular currently used e-cigarette flavors, respectively.

**Conclusions:**

Adult frequent e-cigarette users in the USA who have completely switched from smoking cigarettes to using e-cigarettes are increasingly likely to have initiated e-cigarette use with non-tobacco flavors and to have transitioned from tobacco to non-tobacco flavors over time. Restricting access to non-tobacco e-cigarette flavors may discourage smokers from attempting to switch to e-cigarettes.

## Background

Tobacco harm reduction (THR) products and policies aim to prevent or reduce harm by promoting substitution of combustible tobacco with less hazardous non-combustible sources of nicotine to smokers who are unable or unwilling to quit smoking in response to conventional tobacco control measures [1]. Electronic cigarettes (e-cigarettes)—hand-held devices that use battery power to heat a solution of propylene glycol, glycerol and often flavorings and nicotine, to produce an aerosol that the user inhales—have, in several countries, rapidly grown in popularity among adults as an alternative to smoking conventional cigarettes [2–6]. E-cigarettes are now the most popular assisted method of quitting smoking in the USA, used in 35% of smokers’ most recent quit attempts. [7] By comparison, nicotine patches or gums were used in 25% of most recent quit attempts.

The ability to inhale e-cigarette vapor aerosol in a vast and growing variety of “characterizing flavors”—a distinguishable taste or aroma, other than the taste or aroma of tobacco—is thought to be a major feature accounting for the appeal of e-cigarettes to adult smokers as an alternative to continuing to smoke cigarettes. An estimated 7700 unique e-liquid flavors were available for purchase in 2014, comprising an assortment of fruit, sweet, candy, dessert, food, and drink flavors. [8] Under the Food and Drug Administration’s (FDA) final deeming rule, decisions as to whether authorizing marketing orders for flavored e-cigarette products would be appropriate for the protection of the public health must be based on a weighing of the risks and benefits to both users and non-users of tobacco products. FDA Commissioner, Scott Gottlieb, has stated “it possible for flavors to do both harm and good… On this issue, we see two sides – on the one hand, we need to know the role that flavors, including menthol, play in attracting youth to initiate tobacco use. But on the other hand, we also need to know whether…certain flavors may help adult cigarette smokers switch to potentially less harmful forms of nicotine delivery such as e-cigarettes” [9]. To this end, on March 21, 2018, FDA issued an advance notice of proposed rulemaking (ANPRM) to obtain information related to the role that flavors play in the population’s use of tobacco products. This ANPRM is seeking data, research results, comments, and other information about, among other things, the extent to which certain flavors may attract youth to initiate use of a tobacco product and the extent to which certain flavors may help adult cigarette smokers quit or reduce cigarette use and switch to potentially less harmful products. FDA is seeking this information to inform regulatory actions that FDA might take with respect to flavored tobacco products under the Federal Food, Drug, and Cosmetic Act (“FD&C Act”), as amended by the Tobacco Control Act.

Evidence from cross-sectional surveys of nationally representative samples of US adults and non-probabilistic surveys of dedicated e-cigarette users suggests that smokers tend to initiate e-cigarette use with tobacco-flavored e-cigarettes but transition to exclusive or predominant use of non-tobacco flavored products—particularly fruit, sweet, and dessert flavors—with increased frequency and duration of e-cigarette use [10, 11]. Data from the Population Assessment of Tobacco and Health (PATH) Study shows that the majority of daily e-cigarette users were currently using non-tobacco flavors and were significantly more likely than moderate and infrequent e-cigarette users to have initiated e-cigarette use with a non-tobacco flavor [10]. At the same time, daily e-cigarette use was associated with higher odds of being a former smoker. Another study found that most former smoking e-cigarette users initiated e-cigarette use with non-tobacco flavors, while initiation with tobacco flavors was more common for dual users [11]. These data may indicate that smokers who initiate e-cigarette use with a non-tobacco-flavored e-cigarette are more likely to become daily e-cigarette users, and in turn, more likely to quit smoking.

A separate analysis of PATH data found that young adult (aged 18–34) cigarette smokers at wave 1 (2013/14) who were using one non-tobacco/menthol flavor or multiple non-tobacco/menthol flavors in an e-cigarette at wave 2 (2014/15) were 2.5 and 3 times more likely to have quit or reduced smoking in the past year, respectively, compared to non-e-cigarette users [12]. Dedicated e-cigarette users who are also former smokers report that switching between flavors within the same day is common and that regular use of multiple e-liquid flavors was associated with significantly higher odds of having quit smoking [13]. E-cigarette flavor preferences also appear to vary by age and smoking status. In a nationally representative survey, young US adult (18–29 years) and older US adult (≥ 30 years) former smokers who had become exclusive e-cigarette users were significantly more likely than dual users of conventional cigarettes and e-cigarettes to have initiated e-cigarette use with a non-tobacco flavor (65.7 vs. 47.3%) [11]. Both former-smoking exclusive e-cigarette users and dual users reported significantly higher rates of current use of a non-tobacco-flavor—72.5 and 72.9%, respectively—compared to initiation, suggesting adult e-cigarette users gravitate towards the use of non-tobacco flavors as e-cigarette use continues, with only around one in ten adult current e-cigarette users using tobacco flavored e-cigarettes. Younger adults were significantly more likely to be currently using fruit (74%) and candy and dessert (50%) flavors than were older adults (47 and 27%, respectively). Older adults were significantly more likely than younger adults to be using tobacco-flavored e-cigarettes (13 vs. 1%). Additionally, exclusive e-cigarette users were more likely than dual users to endorse “liking of flavors” as a reason for current e-cigarette use (69.8 vs. 48.9%), suggesting the use of non-tobacco flavors may be positively associated with smokers’ likelihood of transitioning to exclusive e-cigarette use.

Despite evidence of a potential role of non-tobacco e-cigarette flavors in helping adults to quit or reduce cigarette smoking, the same concerns that led the US Congress to ban the sale of cigarettes with characterizing flavors in 2009 now exist for e-cigarettes. In particular, concerns have been raised that fruit and sweet e-liquid flavors will attract youth and non-smokers to e-cigarette use, that use of flavored e-cigarettes will habituate youth to the effects of nicotine, and in turn, youth who would otherwise not have smoked in the absence of flavored e-cigarettes will “graduate” to use of more harmful tobacco products, such as cigarettes, that deliver nicotine more efficiently [14]. These concerns are borne from data that show the majority of youth and young adults who have ever tried an e-cigarette started their use with fruit or sweet flavors rather than a tobacco flavor and that rates of use of flavored tobacco products are higher among youth and young adults than among older adults [15–18]. Other research suggests adolescents’ intentions to try using e-cigarettes are linked to the availability of non-tobacco flavors [19, 20]. Concerns have also been raised about the long-term health effects of inhalation of e-cigarette flavorings [13].

While the Tobacco Control Act does not ban the sale of flavored e-cigarette products, it does not pre-empt state and local governments from restricting or banning the sale of these products. The city of Chicago, for example, in 2013 adopted an ordinance that prohibited the sale of flavored tobacco products, including menthol cigarettes and e-cigarettes, within 500 ft of schools [21]. In June 2017, the city of San Francisco became the first US city to sign into law an ordinance prohibiting the sale of all flavored tobacco products, including all flavors of e-liquid except tobacco flavor [22]. This ordinance came into effect in April 2018, though the fate of the ordinance will be decided by San Francisco voters after a petition drive by R.J. Reynolds Tobacco Company gained enough signatures to put the ordinance on a ballot in San Francisco County on June 5, 2018 [23]. Both the Chicago and San Francisco ordinances were adopted as precautionary measures in the absence of an established scientific basis for estimating that restricting the use and availability of e-cigarettes in characterizing flavors would be appropriate for the benefit and protection of the public health. The ANPRM recently announced by FDA represents the first steps to establishing a scientific basis for the regulation of flavors in tobacco products, including e-cigarettes, that weighs the risks and benefits of flavored e-cigarettes to the population as a whole, including their appeal to, use by, and effect of the tobacco use behaviors of current users, former users, and non-users of tobacco products.

The present study sought to obtain information on the flavor preferences of frequent e-cigarette users for two main reasons. First, frequent e-cigarette users should be at greater risk of being harmed by and addicted to e-cigarettes compared to infrequent users and former users. Understanding the extent to which different flavors are used by this sub-group, and the effect that frequent use of different flavors has on cigarette smoking, is therefore of great importance to assessing the likelihood that frequent use of different e-cigarette flavors is likely to add or reduce risk of harm to users. Second, much of what is known about the flavor preferences of e-cigarette users is based on surveys of nationally representative samples that are largely comprised of infrequent e-cigarette users. The extent to which the flavor preferences of infrequent e-cigarette users apply to frequent e-cigarette users is unclear.

Assessing the first use and current use of flavored e-cigarettes and e-liquids among current smokers, former smokers, and never smokers who currently use e-cigarettes on a frequent basis can therefore help inform the potential population health impact of these products. The purpose of the present study was to assess the first e-cigarette flavor and current e-cigarette flavors used by a large non-probabilistic sample of adult frequent users of e-cigarettes in the USA.

## Methods

### Recruitment materials

A study invitation called for individuals aged 18 years or older, living in the USA, who have ever used an e-cigarette, even a single puff, to complete a 20-min online survey about their current and past use of e-cigarettes and conventional tobacco cigarettes. “E-cigarette use” was defined as “use of any cigalike, pre-filled device, eGo-style vaping device, Mod-style vaping device, or advanced personal vaporizer.” The study invitation clarified that people who smoke cigarettes, used to smoke cigarettes, or have never smoked cigarettes were equally welcome to complete the survey. The invitation contained a web-link to the survey homepage. Data collection ran from May 1 to June 30 2016. No financial or other incentive was offered in exchange for participation. A favorable ethical opinion of this study was given by the University of Strathclyde Research Ethics Committee.

### Recruitment strategies

The population of interest in this study was adults (aged 18 years and older) living in the USA who were frequent users of an e-cigarette or personal vaporizer, with “frequent use” defined as use on at least 20 of the past 30 days. It was hypothesized that frequent e-cigarette use would be the predominant use frequency among adults who are actively engaged in discussion, advocacy, and events related to vaping and vapor products. Participant recruitment was therefore conducted in two ways.

First, a survey invitation was emailed to members of four US-based organizations with large memberships of e-cigarette/vapor product users: Consumer Advocates for Smoke-Free Alternatives Association (CASAA), Smoke-Free Alternatives Trade Association (SFATA), the American Vaping Association (AVA), and Not Blowing Smoke (NBS). According to their websites, the broad mission of these organizations is to raise public awareness and education around tobacco harm reduction, provide smokers and non-smokers with scientific information about the relative risks associated with use of different tobacco and nicotine-containing products, and advocate for adults who want to use vapor products as an alternative to continuing to use combustible tobacco products. The study investigators did not request, have sight of, or have access to any organization’s membership list. Reminder emails were sent after 7 and 14 days.

Second, the survey invitation was posted by account administrators to the social media accounts (e.g., Facebook, Twitter, Instagram) belonging to CASAA, SFATA, AVA, and NBS and to a number of social media platforms (e.g., Facebook, Twitter, Reddit, Instagram) and e-cigarette discussion forums (e.g. *E-Cigarette Forum, Planet of the Vapes*) that are dedicated to vaping-related discussion.

### Survey procedure

Clicking the link in the study advertisement routed the individual to the study information page, which described the purpose of the survey, the names and contact details of the study investigators, information about who is eligible to take part and how survey data will be used, assurances of participant anonymity and confidentiality, and the source of funding for this study. Individuals who satisfied eligibility criteria and gave informed consent to participate began the survey. Participants were asked to answer the survey as fully as possible but informed that they were free to skip any questions they did not wish to answer. Duplicate survey entries were identified as either those with the same e-mail address or those with the same IP address, state, gender, and age.

### Survey measures

#### Chronology of initiation, cessation, and re-initiation of cigarette smoking and e-cigarette use

Questions assessed the prevalence of ever use, current use, and frequent use of combustible cigarettes and e-cigarettes, and the chronological order in which participants had started, stopped, and re-started use of combustible cigarettes and e-cigarettes. On the basis of responses to these questions, participants were first classified as either (i) a frequent e-cigarette user—used an e-cigarette on ≥ 20 of the past 30 days–or (ii) an infrequent e-cigarette user–used an e-cigarette on 0–19 of the past 30 days.

Frequent e-cigarette users were then further classified as one of the six types of cigarette smoker. These six classifications, referred to as *Tobacco Use Pathway Groups* (TUPs), represent all the transitions an individual may conceivably make between cigarette smoking and frequent e-cigarette use and the chronological order in which these transitions occur. The criteria used to classify frequent e-cigarette users into each TUP are shown in Table 1.

**Table 1:** Criteria for classification of frequent e-cigarette users into six Tobacco Use Pathway (TUP) groups

Num.	TUP group label	TUP group definition
1	Switchers	A lifetime smoker (≥ 100 cigarettes smoked) who smoked cigarettes regularly *prior* to initiating e-cigarette use *and* had quit smoking completely (no smoking in the past 30 days) at the time of survey *and* was a frequent e-cigarette user at the time of survey.
2	Dual users of cigarettes and e-cigarettes	A lifetime smoker (≥ 100 cigarettes smoked) who smoked cigarettes regularly *prior* to initiating e-cigarette use *and* was a current smoker (smoked in the past 30 days) *and* a frequent e-cigarette user at the time of survey.
3	Former smoker-turned-dual users	A lifetime smoker (≥ 100 cigarettes smoked) who had quit smoking completely *prior* to initiating e-cigarette use *and* was a current smoker (smoked in the past 30 days) *and* a frequent e-cigarette user at the time of survey.
4	Former-smoker e-cigarette users	A lifetime smoker (≥ 100 cigarettes smoked) who had quit smoking completely *prior* to initiating e-cigarette use *and* has not re-initiated regular smoking *since* initiating e-cigarette use *and* was a frequent e-cigarette user at the time of survey.
5	Never-smoker-turned-dual users	A lifetime never-smoker (< 100 cigarettes smoked) who was not smoking at the point of his/her first use of an e-cigarette *and* was a current smoker *and* a frequent e-cigarette user at the time of survey.
6	Never-smoker e-cigarette users	A lifetime never-smoker (< 100 cigarettes smoked) who was not smoking at the point of his/her first use of an e-cigarette *and* was not a current smoker at the time of survey *and* was a frequent e-cigarette user at the time of survey.

### Demographic characteristics

Questions assessed participants’ age, gender, census region, educational attainment, and employment status.

### First and current device, flavor, and nicotine concentration

Participants were asked the format, flavor, and nicotine concentration of the first e-cigarette/e-liquid they purchased for personal use, the format of the device used most often now, currently used e-cigarette/e-liquid flavors, and the nicotine concentration of e-cigarettes/e-liquids used most often now. Participants reported the approximate time of their first e-cigarette purchase; for the analysis, data were categorized as “≥ 5 years ago” (coded as 1), “3 to 5 years ago” (coded as 2), “1 to 3 years ago” (coded as 3), and “less than 12 months ago” (coded as 4). Questions also assessed weekly consumption of disposable e-cigarettes, pre-filled cartridges, or volume (mLs) of e-liquid, depending on the format of the device currently used most often. Current users of rechargeable devices with a refillable tank were also asked the coil resistance and typical wattage at which they used their current device, if applicable.

### Statistical analysis

All analyses were performed in four exclusive smoking status groups (switchers, dual users, former smoker e-cigarette users, and never smoker e-cigarette user); two groups (former smokers-turned-dual users and never smokers-turned-dual users) were excluded from analyses due to extremely low cell sizes. Descriptive statistics—mean (SD) or number (%)—are reported for demographic variables. Chi-square (*χ*^2^) tests compared the prevalence of first e-cigarette flavor purchased, separately for each time period of first e-cigarette purchase. Cross-tabulations and chi-square tests compared the prevalence of first e-cigarette purchases that were flavored to taste like “tobacco” versus “fruit/fruit beverage” between different TUP groups. Logistic regression analyses were conducted to evaluate the association between the first e-cigarette purchases that were flavored to taste like “tobacco” versus “fruit/fruit beverage” and TUPs and time period of first e-cigarette purchase. Similar analyses were conducted for current use of tobacco and fruit/fruit beverage flavored e-cigarettes. The analyses were adjusted for age and gender, and the time period of first e-cigarette purchase was entered in the model as an ordinal scale variable to estimate the change in odds of tobacco and fruit/fruit beverage flavored first e-cigarette purchases associated with each change in time period from ≥ 5 years (reference group: coded as 1) to less than 12 months ago (coded as 4). A *p* value < 0.05 was considered statistically significant, and all analyses were conducted in SPSS v24.

## Results

### Participants

After removal of 286 duplicate entries, 758 entries from individuals not living in the USA (Canada *n* = 289; UK *n* = 291; country of residence not specified *n* = 178), and 396 entries from individuals who did not indicate where they found out about the survey, a sample of 22,411 US-based, adult (≥18 years), ever-users of e-cigarettes was retained for preliminary analysis. Within this sample, 20,836 (92.9%) respondents were frequent e-cigarette users at the time of survey. The distribution of these 20,836 respondents across the six TUPs is shown in Table 2. Demographic characteristics of the frequent e-cigarette users, stratified by Tobacco Use Pathway Group, are summarized in Table 3. Demographic characteristics are not reported for former smokers-turned-dual users or never smokers-turned-dual users due to low cell sizes. Thus, the analyses of flavor preferences were confined to 20,676 participants.

**Table 2:** Classification of 20,836 US adult frequent e-cigarette users into six Tobacco Use Pathway Groups (TUPs)

Num.	TUP group	*N*	% of total sample	% of frequent EC users
1	Switchers	15,807	70.5	75.9
2	Dual users of cigarettes and e-cigarettes	1330	5.9	6.4
3	Former smoker-turned-dual users	129	0.6	0.6
4	Former-smoker e-cigarette users	2483	11.1	11.9
5	Never-smoker-turned-dual users	31	0.1	0.1
6	Never-smoker e-cigarette users	1056	4.7	5.1

**Table 3:** Demographic characteristics of 20,676 US adult frequent e-cigarette users classified into four Tobacco Use Pathway (TUP) groups

	Switchers (*n* = 15,807)	Dual users (*n* = 1330)	Former smoker e-cigarette users (*n* = 2483)	Never smoker e-cigarette users (*n* = 1056)	Total (*n* = 20,676)
Variable	*N* (%)	*N* (%)	*N* (%)	*N* (%)	*N* (%)
Age group					
18–25	1714 (10.8)	264 (19.8)	587 (23.6)	579 (54.8)	3144 (15.2)
26–35	4165 (26.4)	378 (28.4)	784 (31.6)	199 (18.8)	5526 (26.7)
36–45	4382 (27.7)	305 (22.9)	543 (21.9)	118 (11.2)	5348 (25.9)
46–55	3193 (20.2)	237 (17.8)	336 (13.5)	92 (8.7)	3858 (18.7)
56–65	1910 (12.1)	124 (9.3)	192 (7.7)	58 (5.5)	2284 (11.0)
66–75	421 (2.7)	19 (1.4)	41 (1.7)	9 (0.9)	490 (2.4)
75+	19 (0.1)	3 (0.2)	0 (0.0)	1 (0.1)	23 (0.1)
Total	15,804 (100.0)	1330 (100.0)	2483 (100.0)	1056 (100.0)	20,673 (100.0)
Gender					
Male	11,173 (71.0)	904 (68.1)	1866 (75.5)	806 (76.9)	14,749 (71.6)
Female	4513 (28.7)	420 (31.6)	594 (24.0)	234 (22.3)	5761 (28.0)
Transgender	51 (0.3)	4 (0.3)	12 (0.5)	8 (0.8)	75 (0.4)
Total	15,737 (100.0)	1328 (100.0)	2472 (100.0)	1048 (100.0)	20,585 (100.0)
Census region-division					
Northeast	2511 (15.8)	228 (17.1)	470 (18.9)	163 (15.5)	3372 (16.3)
New England	686 (4.3)	51 (3.8)	109 (4.4)	43 (4.1)	889 (4.3)
Middle Atlantic	1825 (11.5)	177 (13.3)	361 (14.5)	120 (11.4)	2483 (12.0)
Midwest	3710 (23.5)	355 (26.7)	547 (22.0)	250 (23.7)	4862 (23.6)
East North Central	2547 (16.1)	254 (19.1)	408 (16.4)	174 (16.5)	3383 (16.4)
West North Central	1163 (7.4)	101 (7.6)	139 (5.6)	76 (7.2)	1479 (7.2)
South	6161 (39.0)	469 (26.7)	857 (34.5)	337 (31.8)	7824 (37.8)
South Atlantic	3074 (19.4)	250 (18.8)	451 (18.2)	162 (15.3)	3937 (19.0)
East South Central	1241 (7.9)	95 (7.1)	162 (6.5)	71 (6.7)	1569 (7.6)
West South Central	1846 (11.7)	124 (9.3)	244 (9.8)	104 (9.8)	2318 (11.2)
West	3085 (21.8)	251 (18.9)	522 (21.1)	259 (24.6)	4117 (19.9)
Mountain	1195 (7.6)	100 (7.5)	200 (8.1)	83 (7.9)	1578 (7.6)
Pacific	1890 (12.0)	151 (11.4)	322 (13.0)	176 (16.7)	2539 (12.3)
No answer	340 (2.2)	27 (2.0)	87 (3.5)	47 (4.5)	501 (2.4)
Total	15,807 (100.0)	1330 (100.0)	2483 (100.0)	1056 (100.0)	20,676 (100.0)
Employment (hours paid work pw)					
35+ hours	11,014 (69.9)	824 (62.1)	1718 (69.5)	549 (52.2)	14,105 (68.5)
15–35 h	1320 (8.4)	142 (10.7)	263 (10.6)	231 (22.0)	1956 (9.5)
0–15 h	320 (2.0)	47 (3.5)	55 (2.2)	50 (4.8)	472 (2.3)
0 h	3092 (19.6)	313 (23.6)	435 (17.6)	221 (21.0)	4061 (19.7)
Total	15,746 (100.0)	1326 (100.0)	2471 (100.0)	1051 (100.0)	20,594 (100.0)
Highest education level					
Less than high school	77 (0.5)	11 (0.8)	17 (0.7)	5 (0.5)	110 (0.5)
Some high school, no diploma	380 (2.4)	50 (3.8)	81 (3.3)	44 (4.2)	555 (2.7)
GED	1019 (6.5)	107 (8.1)	160 (6.5)	26 (2.5)	1312 (6.4)
High school graduate, diploma	2944 (18.6)	270 (20.3)	619 (25.0)	347 (32.9)	4180 (20.2)
Some college, no degree	5496 (34.8)	462 (34.8)	812 (32.8)	336 (31.9)	7106 (34.4)
Associate degree—voc./occup.	1736 (11.0)	119 (9.0)	269 (10.9)	83 (7.9)	2207 (10.7)
Associate degree—academic	1070 (6.8)	78 (5.9)	149 (6.0)	63 (6.0)	1360 (6.6)
Bachelor’s degree	2325 (14.7)	178 (13.4)	287 (11.6)	105 (10.0)	2895 (14.0)
Master’s degree	513 (3.2)	37 (2.8)	64 (2.4)	35 (3.3)	649 (3.1)
Professional school degree	131 (0.8)	9 (0.7)	10 (0.4)	7 (0.7)	157 (0.8)
Doctorate degree	95 (0.6)	8 (0.6)	11 (0.4)	3 (0.3)	117 (0.6)
Total	15,786 (100.0)	1329 (100.0)	2479 (100.0)	1054 (100.0)	20,648 (100.0)
Vape advocacy group membership					
CASAA	10,442 (66.1)	694 (52.2)	1620 (65.2)	500 (47.3)	13,256 (64.1)
SFATA	1512 (9.6)	60 (4.5)	313 (12.6)	107 (10.1)	1992 (9.6)
NBS	3126 (19.8)	221 (16.6)	640 (25.8)	302 (28.6)	4289 (20.7)
AVA	1432 (9.1)	108 (8.1)	307 (12.4)	117 (11.1)	1964 (9.5)

### E-cigarette device format currently used

The majority of frequent e-cigarette users in each TUP group reported current main use of a rechargeable e-cigarette device with a refillable tank/reservoir (switchers = 85.7%; dual users = 87.9%; former smoker e-cigarette users = 81.6%; and never smoker e-cigarette users = 79.8%). Current main use of an e-cigarette kit (rechargeable with pre-filled cartridges) was less than 1% in all TUP groups, and current main use of disposable e-cigarettes was essentially zero in all TUP groups.

### First e-liquid flavor purchased

**Fig. 1:**
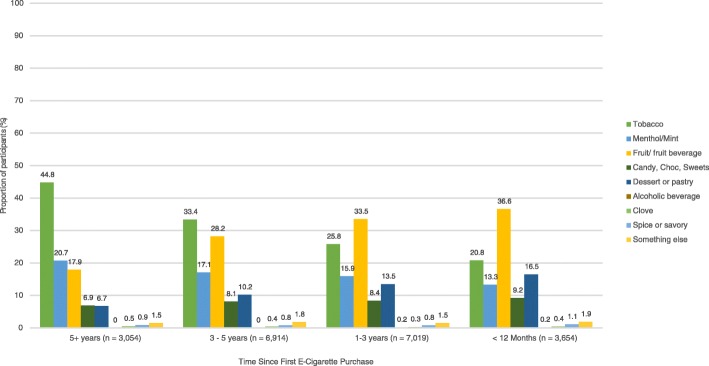
Flavor of first e-cigarette purchased by time since first e-cigarette purchase: frequent e-cigarette users (*n* = 20,641/20,676)

The lengths of time since participants made their first e-cigarette are summarised in Table 4. Chi-square tests indicated statistically significant differences in the prevalence of first e-cigarette flavor purchased at all time points of e-cigarette use initiation (*p* <  0.001 for all). Tobacco flavor was the most popular first flavor purchased by those who initiated e-cigarette use ≥ 5 years ago and between 3 and 5 years ago but declined among those who initiated e-cigarette use 1–3 years ago and in the past 12 months (Fig. 1). First e-liquid purchases that were menthol/mint-flavored had also steadily declined, from being ranked second most common first flavor prior to 2011 to being ranked fourth in the past 12 months. Since 2013, fruit-flavored e-liquids have replaced tobacco-flavored e-liquids as the most popular flavors with which participants had initiated e-cigarette use. The proportion of first e-cigarette purchases that were dessert/pastry-flavored had also increased consistently, from being ranked fifth most common first flavor prior to 2011 to being ranked third in the past 12 months (Table 4).

**Table 4:** Time since first e-cigarette purchase, stratified by Tobacco Use Pathway (TUP) group

TUP group	Time since first e-cigarette purchase				
	≥ 5 years	3–5 years	1–3 years	< 12 months	Total
	*N* (%)	*N* (%)	*N* (%)	*N* (%)	*N*
Switchers	2576 (16.3)	5567 (35.3)	5230 (33.1)	2406 (15.2)	15,779
Dual users	142 (10.7)	334 (25.1)	483 (36.3)	370 (27.8)	1329
Former smoker e-cigarette users	281 (11.3)	788 (31.8)	901 (36.3)	510 (20.6)	2480
Never smoker e-cigarette users	55 (5.2)	225 (21.4)	405 (38.4)	368 (34.9)	1053

Time since first e-cigarette purchase recorded in June 2016

**Fig. 2:**
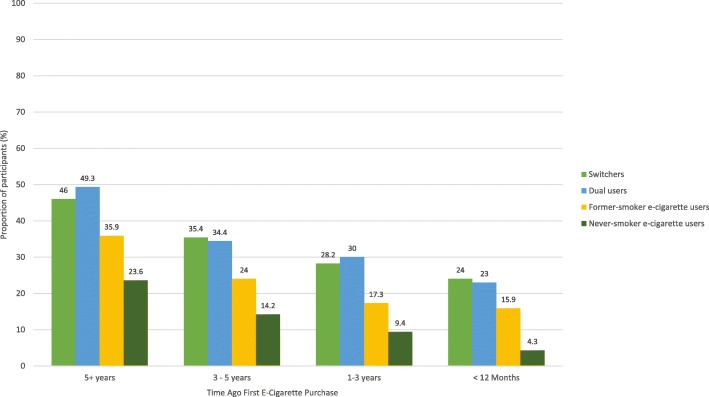
Proportion of e-cigarette first purchases that were flavored to taste like tobacco stratified by time since first e-cigarette purchase and Tobacco Use Pathway (TUP) group

**Fig. 3:**
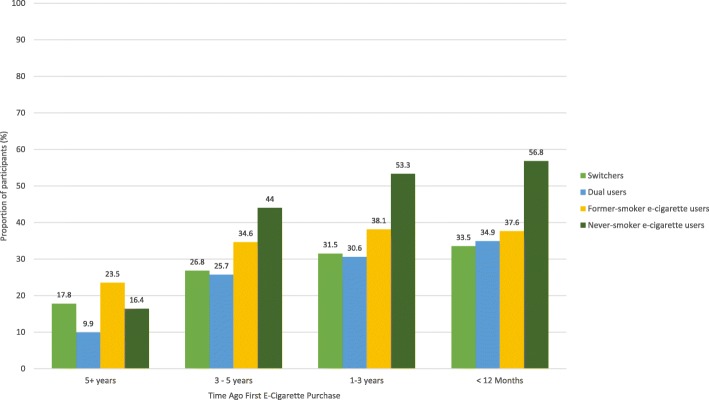
Proportion of first e-cigarette purchases that were flavored to taste like fruit/fruit beverage stratified by time since first e-cigarette purchase and Tobacco Use Pathway (TUP) group

Figures 2 and 3 show that, in each of the four TUP groups, the proportion of first e-liquid purchases that were tobacco-flavored had declined over time, while the proportion of first e-liquid purchases that were fruit-flavored had increased over time. The lowest prevalence of tobacco-flavored first e-cigarette purchases was observed among former smoker e-cigarette users and never smoker e-cigarette users (Fig. 2). Since 2011, the highest rate of fruit-flavored first e-cigarette purchases has consistently been among never smoker e-cigarette users.

Chi-square tests indicated a statistically significant difference in the prevalence of tobacco flavor initiation between TUP groups overall (*p* <  0.001) and statistically significant differences between TUP groups within each time period of e-cigarette use initiation (all ps <  0.001). The proportions of switchers and dual users who initiated e-cigarette use with a tobacco-flavored product were similar in all four time periods. For fruit/fruit beverage flavors, statistically significant differences were observed overall (*p* <  0.001) and for each time point of e-cigarette use initiation (*p* = 0.006 for > 5 years, *p* <  0.001 for all other time points). The proportions of switchers and dual users who initiated e-cigarette use with a fruit-flavored e-cigarette were similar except among those who initiated e-cigarette use 5 or more years ago.

Logistic regression analysis (Table 5) showed that odds of a tobacco-flavored first e-cigarette purchase reduced with the recency of the first e-cigarette purchase, from “> 5 years ago” to “in the past 12 months.” Additionally, switchers and dual users were each four times more likely than never smoker e-cigarette users to have initiated e-cigarette use with a tobacco-flavored product; former smoker e-cigarette users were 2.3 times more likely than never smoker e-cigarette users to have initiated e-cigarette use with a tobacco-flavored product. Conversely, the odds of initiating e-cigarette use with fruit-flavored product increased with the recency of the first e-cigarette purchase. Switchers, dual users, and former smoker e-cigarette users were all significantly less likely than never smoker e-cigarette users to have initiated e-cigarette use with fruit-flavored products.

**Table 5:** Logistic regression analysis of the association between first e-cigarette purchases that were tobacco-flavored and fruit/fruit beverage-flavored and Tobacco Use Pathway Group and time of first e-cigarette purchase

Variable	*B*	OR	95% CI	*p*
Tobacco-flavored FEP				
TUP group				
Never smoker e-cigarette users (referent)				
Switchers	1.39	4.03	3.26–4.97	< 0.001
Dual users	1.42	4.14	3.26–5.26	< 0.001
Former smoker e-cigarette users	0.85	2.33	1.85–2.93	< 0.001
Time of FEP				
> 5 years (referent)				
< 12 months	− 1.04	0.35	0.32–0.40	< 0.001
1–3 years	− 0.80	0.45	0.41–0.49	< 0.001
3–5 years	− 0.46	0.63	0.58–0.69	< 0.001
Fruit/fruit beverage-flavored FEP				
TUP group				
Never smoker e-cigarette users (referent)				
Switchers	− 0.85	0.43	0.38–0.49	< 0.001
Dual users	− 0.90	0.41	0.34–0.48	< 0.001
Former smoker e-cigarette users	− 0.55	0.58	0.50–0.67	< 0.001
Time of FEP				
> 5 years (referent)				
< 12 months	0.89	2.43	2.16–2.72	< 0.001
1–3 years	0.79	2.21	1.99–2.46	< 0.001
3–5 years	0.57	1.76	1.59–1.96	< 0.001

*TUP* Tobacco Use Pathway, *FEP* First E-cigarette purchase

### Currently used e-liquid flavors

**Fig. 4:**
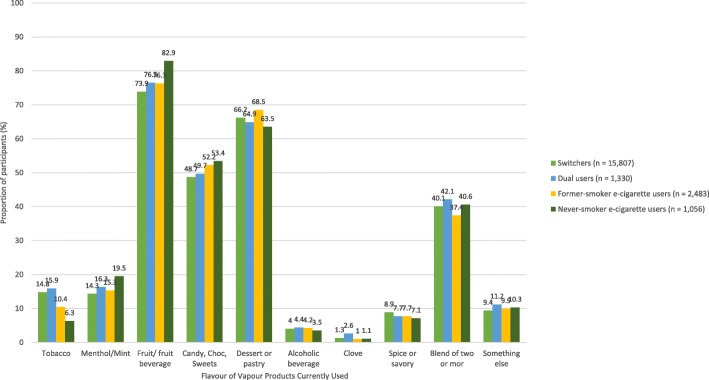
E-cigarette/e-liquid flavors currently used by 20,676 US adult frequent e-cigarette users stratified by Tobacco Use Pathway (TUP) group

The most popular currently used e-cigarette flavors within each TUP group were fruit/fruit beverage followed by dessert/pastry and candy/chocolate/sweets (Fig. 4). Approximately 4 in 10 participants were currently using e-cigarettes containing a blend of two or more flavors. Rates of current use of tobacco-flavored e-liquids were low in all four groups. Once the most popular flavors at the point of first purchase of an e-cigarette, tobacco and menthol/mint-flavored e-liquids were the fifth and sixth most popular currently used flavors by switchers and dual users.

**Fig. 5:**
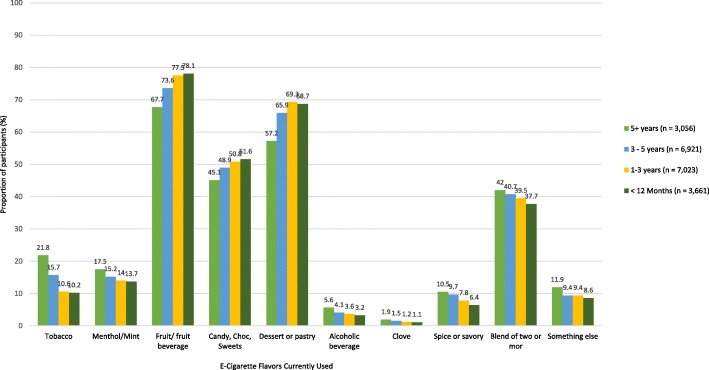
E-cigarette/e-liquid flavors currently used by 20,611 US adult frequent e-cigarette users stratified by time since first e-cigarette purchase

The highest rate of current use of tobacco-flavored e-liquid was reported by those who initiated e-cigarette use ≥ 5 years ago; the lowest rate of current use of tobacco flavor was reported by those who initiated e-cigarette use in the past 12 months (Fig. 5). The highest rate of current use of fruit/fruit beverage flavors was among those who initiated e-cigarette use in the past 12 months; the lowest rate of current use of fruit/fruit beverage flavors was among those who initiated e-cigarette use ≥ 5 years ago. A similar effect of time since first e-cigarette purchase was found for current use of dessert/pastry flavors and for candy/chocolate/sweets flavors.

Logistic regression analysis (Table 6) showed that, compared to first purchases made > 5 years ago, odds of current use of tobacco-flavored e-liquid decreased with recency of first e-cigarette purchase, with the lowest rate of current use of tobacco-flavored e-liquid observed among those who purchased their first e-cigarette in the past 12 months. As was observed for tobacco-flavored first e-cigarette purchases, switchers, dual users, and former smoker e-cigarette users all had significantly higher odds of current use of tobacco-flavored e-liquid compared to never smoker e-cigarette users, though these odds for current use of tobacco-flavored products were considerably smaller than the odds for initiating use with a tobacco-flavored product. Conversely, odds of current use of fruit/fruit beverage flavors were observed to increase with the recency of a participant’s first e-cigarette purchase, with the highest odds of current use of fruit-flavored product observed among those who purchased their first e-cigarette in the past 12 months. Though the odds of current use of a fruit-flavored product had increased over time in all groups, switchers, dual users, and former smoker e-cigarette users were significantly less likely than never smoker e-cigarette users to be current users of fruit-flavored products.

**Table 6:** Logistic regression analysis of the association between current use of tobacco-flavored e-liquids and fruit/fruit beverage-flavored e-liquids and Tobacco Use Pathway Group and time of first e-cigarette purchase

Variable	*B*	OR	95% CI	*p*
Current use of tobacco flavor				
Never smoker e-cigarette users (referent)				
Switchers	0.78	2.18	1.69–2.81	< 0.001
Dual users	0.97	2.63	1.97–3.51	< 0.001
Former smoker e-cigarette users	0.43	1.54	1.16–2.03	< 0.001
Time of FEP				
> 5 years (referent)				
< 12 months	− 0.84	0.43	0.38–0.50	< 0.001
1–3 years	− 0.82	0.44	0.39–0.49	< 0.001
3–5 years	− 0.39	0.68	0.61–0.76	< 0.001
Current use of fruit-fruit beverage flavor				
Never smoker e-cigarette users (referent)				
Switchers	−0.45	0.64	0.54–0.75	< 0.001
Dual users	−0.36	0.70	0.57–0.86	< 0.001
Former smoker e-cigarette users	−0.35	0.70	0.59–0.85	< 0.001
Time of FEP				
> 5 years (referent)				
< 12 months	0.49	1.62	1.46–1.81	< 0.001
1–3 years	0.47	1.61	1.46–1.77	< 0.001
3–5 years	0.28	1.32	1.20–1.45	< 0.001

*TUP* Tobacco Use Pathway, *FEP* First E-cigarette purchase

## Discussion

This study assessed first and current use of tobacco and non-tobacco e-cigarette flavors by a non-probabilistic sample of 20,836 adults in the USA who were using e-cigarettes on a frequent basis, of whom 15,807 (75.9%) had completely switched from smoking cigarettes to using e-cigarettes. Results indicated that adults who have completely switched from smoking cigarettes to using e-cigarettes in the past 5 years are increasingly likely to have initiated e-cigarette use with vapor products not flavored to taste like tobacco. E-cigarette flavors that are not available through cigarettes—particularly fruit, dessert, and pastry flavors—were found to have increasingly replaced tobacco and menthol as the preferred flavors with which adult smokers, former smokers, and never smokers have initiated e-cigarette use.

Between 2011 and 2016, the proportion of first e-cigarette purchases that were flavored to taste like a fruit had almost doubled, while tobacco-flavored first e-cigarette purchases had almost halved. These data suggest a transition in flavor preference at e-cigarette use initiation over time, from tobacco to non-tobacco flavors, which is consistent with data from a US nationally representative survey that found both former-smoking exclusive e-cigarette users and dual users reported significantly higher rates of current use of a non-tobacco-flavor—72.5 and 72.9%, respectively—compared to initiation [11]. The proportions of switchers and dual users who initiated e-cigarette use with fruit flavors did not significantly differ among those who initiated e-cigarette use after 2011. This finding contrasts with the data from a US nationally representative survey that showed former smokers who had become exclusive e-cigarette users were significantly more likely than dual users of conventional cigarettes and e-cigarettes to have initiated e-cigarette use with a non-tobacco flavor (65.7 vs. 47.3%) [11]. One potential explanation for these contrasting findings is the different frequencies of e-cigarette use in the samples analyzed; the majority of individuals in the US nationally representative sample were likely to have been infrequent e-cigarette users—i.e., used an e-cigarette on less than 20 of the past 30 days—whereas the present study included only frequent users. The present findings therefore indicate that switchers and dual users were equally likely to have initiated e-cigarette use with fruit-flavored e-cigarettes at any time after 2011, but both have been increasingly likely to have initiated e-cigarette use with a fruit-flavored e-cigarette.

Current e-cigarette use among participants was dominated by use of non-tobacco flavors, mainly fruit/fruit beverage, dessert/pastry, and/or candy/chocolate/sweets flavors. Once the most popular first flavors purchased by switchers and dual users, tobacco and menthol/mint currently rank as the 5th and 6th most commonly used flavors. Comparable odds of current use of fruit/fruit beverage flavor were observed in switchers, dual users, and never smoker e-cigarette users. These findings suggest both that non-tobacco flavors are comparably attractive to smokers who may or may not intend to quit smoking as they are to non-smokers and that non-tobacco flavors are not more strongly associated with dual use (i.e., continuing to smoke) than they are with quitting smoking. Given that the taste of increasingly preferred e-cigarette flavors such as fruits, desserts, and pastries are very different from the taste of a conventional cigarette, the increasing likelihood that adults will initiate e-cigarette use and currently use an e-cigarette use with a non-tobacco flavor could have the beneficial effects of discouraging a return to smoking among adults who switch to e-cigarettes and discouraging non-smoking e-cigarette users from initiating cigarette use.

The positive association observed between the use of fruit flavor and time of first e-cigarette purchase suggests it is likely that future frequent e-cigarette use will be increasingly initiated with the use of non-tobacco flavors, regardless of the individual’s smoking history. Judgements on whether authorizing marketing of flavored e-cigarettes would be appropriate for the benefit and protection of the public health should account for the possibility that adults who have switched completely from smoking cigarettes to using e-cigarettes in non-tobacco flavors may not have attempted to switch to e-cigarettes, or perceived themselves as able to switch, had e-cigarettes only been available in the flavors that are available through conventional cigarettes. A tobacco regulatory policy that maintains adult smokers’ access to the existing market of non-tobacco flavors, and enables manufacturers to innovate existing flavors, could increase the popularity and effectiveness of e-cigarettes as a substitute for cigarette smoking.

A low rate of first use of an e-cigarette that was tobacco-flavored by those who had quit smoking completely *prior* to their first use of an e-cigarette was observed. A possible explanation for this is that these individuals, having already quit smoking completely, wished to resume using nicotine by inhalation but did not wish to taste a tobacco flavor that has, for them, historically functioned as a conditioned stimulus for smoking thoughts and memories, and in turn, cravings. To the extent that this explains part of these former smokers’ reasons for initiating e-cigarette use with non-tobacco flavored e-cigarettes/e-liquids, then restricting access to non-tobacco flavors may remove a method that many former smokers have found effective for attenuating nicotine cravings and preventing relapse to smoking, and so in turn, increase the likelihood that former smokers will resume cigarette smoking to satisfy nicotine cravings.

This study also identified rapid changes in patterns of e-cigarette use over time that highlight the need for policymakers and researchers to continually update their understanding of smokers’ changing preferences for using e-cigarettes of different styles, formats, and performance capabilities. There is also a need for research to better understand smokers’ objections and barriers to switching to e-cigarettes and other proven and potentially less harmful sources of nicotine, and to respond with policies that are more likely to rationalize a switch away from conventional cigarettes, both among those who are open to giving up smoking if offered an appealing alternative to cigarettes and to those who appear to be hardened against ever stopping smoking.

Above all, there is a need to continually study the effect on adult and youth smoking rates of legislation and regulatory frameworks that either maintain or restrict access to a diverse variety of e-cigarette products that reflect users’ heterogeneity of preferences in terms of esthetic appeal (the look and feel of the device), sensory appeal (the taste and smell of vapor), and pharmacological appeal (the speed and efficiency of nicotine delivery).

### Study limitations

The conclusions of this study are limited in several ways. First, the sample is not representative of the general US adult population nor was the study designed or intended to estimate the prevalence or frequency of e-cigarette use and cigarette smoking in the general US adult population. The study aimed to elicit data on patterns of use by a specific sub-group of US adult e-cigarette user—those who are using e-cigarettes on a daily or near-daily basis. The recruitment methods were therefore biased towards outlets where such sub-group of e-cigarette user was most likely to be found, and so conclusions therefore do not represent the flavor preferences or patterns of e-cigarette use of US adults who are using e-cigarettes only experimentally or infrequently, patterns which together account for approximately 79% of all e-cigarette use in the USA [24]The flavor preferences and patterns of e-cigarette use reported by the present sample of frequent e-cigarette users may more closely represent those of the 21.3% of current e-cigarette users in the USA who use e-cigarettes daily [24]. Conclusions about changing flavor preferences are also unlikely to be applicable to US adults who do not frequently use rechargeable, refillable devices, as there is evidence that preferences for tobacco and menthol flavors vary between users of closed-system and open system devices. A consumer representative survey of 2000 US e-cigarette users, for example, found that 84% of users of advanced tanks (refilled with open liquid) currently used non-tobacco flavors compared to 54% of users of rechargeable cigalikes (reloaded with prefilled cartridges) [25]. Users of advanced tanks were significantly less likely than users of rechargeable “cigalikes” to be using tobacco (16 vs 46%) or menthol (18 vs. 25%) flavors and significantly more likely to be using fruit (36 vs. 15%) and sweet (11 vs. 4%) flavors. These self-reported device-specific data closely approximate distributor shipment to retail data for February 2016 [26], which show tobacco and menthol flavors accounted for 67% of the total volume of cartridges shipped to retail in 2016 compared to 50% of the total volume of liquids shipped to retail. Lastly, the study conclusions may not apply to frequent e-cigarette users who do not engage with e-cigarette advocacy groups and online forums.

Another limitation is the study’s reliance on accurate self-reporting of the nature and timing of behaviors that may have occurred up to several years ago. Additionally, the cross-sectional design and non-probabilistic sampling method prevent conclusions about the relative effectiveness of tobacco versus non-tobacco flavored e-cigarettes for producing smoking cessation. Finally, participants in this study were not asked to identify the flavors they were using at the point at which they stopped smoking and so were not asked how frequently they were using each flavor at the point at which they stopped smoking.

## Conclusions

This study identified an increasing popularity of non-tobacco flavors and declining popularity of tobacco flavors by over 15,000 adult frequent e-cigarette users who formerly smoked cigarettes. The findings suggest that access to a variety of non-tobacco flavored e-liquid may be important for encouraging and assisting adults to use e-cigarettes in place of conventional cigarettes. Restricting the availability of non-tobacco flavors could reduce adult smokers’ interest in switching to e-cigarettes or rationalize a return to cigarette smoking among frequent e-cigarette users whose journey towards smoking abstinence started with, progressed to, and is being sustained by frequent use of e-cigarettes containing non-tobacco flavors. A tobacco products regulatory framework that balances adult smokers’ increasingly common preference to try to quit smoking by using e-cigarettes that do not taste like cigarettes, with measures that reduce the appeal and use of e-cigarettes by non-smokers and youth, may accelerate the US progress towards the end of the tobacco smoking epidemic that causes the premature death of approximately 480,000 Americans each year [27].

## Abbreviations

ANPRM: Advanced Notice of Proposed Rule Making
AVA: American Vaping Association
CASAA: Consumer Advocates for Smoke-free Alternatives Association
FD&C: Food, Drug and Cosmetic Act
FDA: US Food and Drug Administration
FEP: First E-cigarette purchase
NBS: Not Blowing Smoke
PATH: Population Assessment of Tobacco and Health
SFATA: Smoke Free Alternatives Trade Association
THR: Tobacco harm reduction
TUP: Tobacco Use Pathway
US: United States
